# Construction of a Single-cell Atlas of Thyroid Cancer

**DOI:** 10.2174/0118715303359688250209090544

**Published:** 2025-04-03

**Authors:** Kaiyu Song, Yaqi Wang, Yuantao Wang, Jiahui Liu, Wenjie Yao, Yongli Chu, Yun Qu, Xicheng Song, Jin Zhou

**Affiliations:** 1 Department of Endocrinology, Yantai Yuhuangding Hospital of Qingdao University, Yantai 264000, Shandong, China;; 2 Department of Otorhinolaryngology, Head and Neck Surgery, Yantai Yuhuangding Hospital, Qingdao University, Yantai, China;; 3 Department of Endocrinology, Binzhou Medical University, Yantai, Shandong, China;; 4 Shandong Provincial Clinical Research Center for Otorhinolaryngologic Diseases, Yantai Yuhuangding Hospital, Qingdao University, Yantai, China;; 5 Yantai Key Laboratory of Otorhinolaryngologic Diseases, Yantai, China;; 6 Department of Gynecology, Yantai Yuhuangding Hospital of Qingdao University, Yantai, China;; 7 Department of Emergency, Yantai Yuhuangding Hospital of Qingdao University, Yantai 264000, Shandong, China;; 8 Key Laboratory of Spatiotemporal Single-Cell Technologies and Translational Medicine, Yantai 264000, Shandong, China

**Keywords:** Thyroid cancer, long non-coding RNA, IGF2BP3, m6A, single-cell sequencing, paraneoplastic tissues, epithelial cells

## Abstract

**Introduction:**

Differentiated thyroid cancer generally has a favorable prognosis; however, the cure rate remains low for patients with metastatic or undifferentiated thyroid cancer. Moreover, this group of patients exhibits diverse responses to different treatments. To address this, single-cell RNA sequencing (scRNA-seq) offers an unbiased approach to reveal the heterogeneity within and between tumor cells. Using, scRNA-seq, we aimed to explore the intricate ecosystem of thyroid cancer, potentially providing novel insights into clinical cancer staging and treatment strategies.

**Methods:**

We conducted a thorough analysis by screening thyroid cancer and paraneoplastic tissues from 20 patients sourced from the Gene Expression Omnibus database. The dataset comprised 11 primary tumor tissues, 6 paraneoplastic tissues, 8 metastatic lymph nodes, and 2 distant metastases of papillary thyroid cancer. Through comprehensive bioinformatic analyses, we constructed a panoramic single-cell atlas of thyroid cancer (THCA).

**Results:**

Our findings revealed significant heterogeneity in gene expression among tumor cells from different patients with THCA, contributing to the development of a comprehensive single-cell landscape. Notably, the long noncoding RNA (lncRNA) gene XIST exhibited higher abundance in anaplastic thyroid cancer (ATC) tumor cells. Additionally, we identified an enriched m6A locus in lncRNA XIST and observed high expression of the m6A “reader” IGF2BP3, as well as low expression of the “encoder” VIRMA. Based on these observations, we hypothesized that IGF2BP3 and VIRMA could augment the expression of lncRNA XIST, thereby promoting the malignant proliferation and invasion of ATC.

**Conclusion:**

By leveraging scRNA-seq technology, our study sheds light on the intricate molecular characteristics of THCA lesions. These findings have the potential to revolutionize our understanding of thyroid cancer pathogenesis and pave the way for innovative therapeutic interventions.

## 1 INTRODUCTION

Thyroid cancer is a prevalent malignancy of the endocrine system [1, 2], and its incidence and mortality rates have been increasing in recent decades [3]. Thyroid cancer arises from follicular epithelial cells or parafollicular cells. Differentiated thyroid cancer, which includes papillary thyroid carcinoma (PTC) and follicular thyroid cancer (FTHCA), accounts for more than 90% of thyroid malignancies [4]. Poorly differentiated thyroid cancer (PDTHCA, 5%) and anaplastic thyroid cancer (ATC, 1%) are less common [4]. PTC, originating from follicular epithelial cells, is the most prevalent type of thyroid cancer. Despite the generally positive prognosis for thyroid cancer, the cure rate for patients with metastases remains low [5]. In particular, ATC is the least curable form, known for its aggressive and lethal nature [6]. It progresses rapidly, and approximately 75% of patients develop distant metastases in the lungs, bones, and brain [7]. ATC has a grim prognosis, accounting for 20-50% of all thyroid cancer-related deaths [8], with a median survival of only 3-6 months. Hence, studying the tumor ecosystem of metastatic papillary thyroid cancer and undifferentiated thyroid cancer is crucial. Additionally, the response to treatments among these patients varies significantly. Therefore, a comprehensive characterization of the entire tumor ecosystem, beyond the current clinical classification based on somatic mutations, is essential.

Conventional RNA sequencing approaches have provided homogeneous analyses of metastatic and undifferentiated thyroid cancer samples, limiting the ability to reveal heterogeneity between tumor components from different patients. In contrast, single-cell RNA sequencing (scRNA-seq) offers a more unbiased depiction of intra- and inter-tumor cell composition [9]. Consequently, we integrated a total of 249,738 single cells from tissue sample data collected from 20 patients in the GEO database, including PTC, ATC, metastatic lymph nodes (MLN) in papillary thyroid cancer, distant metastatic tissue of papillary thyroid cancer (MT), and paracancerous tissue (PCa).

In this study, we aimed to explore the heterogeneity of thyroid cancer in patients and the differences in gene expression among tumor cells under various pathological stages, as well as the corresponding alterations in the tumor microenvironment, from a single-cell perspective. We created a comprehensive map of the single-cell ecosystem of THCA, identified relevant cell clusters, and specifically identified a cell cluster, THCA_XIST_IGF2BP3, which exhibited specific expression in female patients with ATC. Furthermore, we confirmed the involvement of relevant specific genes in signaling pathways and gene regulatory networks (GRN), providing a deeper understanding of the complex ecosystem of thyroid cancer.

## 2 MATERIALS AND METHODS

### 2.1 Data Sources

The THCA scRNA-seq data used in this study were obtained from publicly available datasets in the Gene Expression Omnibus (GEO) database of the National Center for Biotechnology Information (NCBI). Specifically, we utilized the following datasets: GSE148673 [10], GSE184362 [11], and GSE191288 [12]. These datasets provided valuable information on tissue samples from 20 patients, encompassing PTC and ATC, as well as MLN, MT, and PCa. Notably, GSE148673 specifically focused on undifferentiated thyroid cancer; GSE184362 encompassed localized/advanced papillary thyroid cancer, initial treatment/recurrent lymph node, and radioactive iodine (RAI)-refractory distant metastases; while GSE191288 included data on papillary thyroid cancer.

### 2.2 Raw Data Integration

Thyroid cancer-associated single-cell transcriptome data GSE148673 [10], GSE184362 [11] and GSE191288 [12] were obtained from GEO data (https://www.ncbi.nlm.nih.gov/geo/). Data were preprocessed using Integrate Data [13]. The app integrates expression profiling data in either base process (Cell Ranger process) or data frame format after single-cell sequencing downstream and generates single-cell expression profiles (Seurat objects) for subsequent analysis.

### 2.3 SC Transform

To model the expression data of single-cell UMIs, we utilize regularized negative binomial regression to account for variations introduced by sequencing depth. Initially, this method constructs a Generalized Linear Model (GLM) with sequencing depth as the independent variable and UMI counts as the response or dependent variable. Subsequently, regularization, or adjustment, of parameter estimation is conducted based on gene expression. A second round of negative binomial regression is then applied using the regularized parameters. The resulting output of this model (residuals) reflects the standardized expression levels for each gene. Additionally, the SCTransform method learns gene-group-specific factors, tailored for low, medium, and high-expression genes, instead of employing constant factors to standardize all genes. These gene-group factors effectively eliminate technical variability while preserving genuine biological heterogeneity.

### 2.4 Construction of the Single-cell Atlas

The construction of the single-cell atlas was conducted using the RunSeurat application, which utilizes the default parameters of the Seurat package in R [14]. The uniform manifold approximation and projection (UMAP) method was used for dimensionality reduction and visualization of the clustering results, and the results were projected to a two-dimensional image, which was defined as a single-cell atlas. To identify specifically expressed genes for each cell cluster, the FindAllMarkers function from the Seurat package was utilized. A significance threshold of *P* < 0.05 was applied to determine the statistical significance of the identified markers.

### 2.5 Differential Expression Analysis

To explore dysregulated genes in THCA, we used the Limma package [1, 15] and *p* < 0.05 and |log fold change (logFC)|>0.5 were considered significant.

### 2.6 Cell Subpopulation Analysis

This study performed a subcluster analysis of each cell type using Seurat to explore the biological functions of disease-specific subgroups. According to differentially expressed genes, the Seurat package enables further clustering of a cell type and identification of subpopulations. Marker genes expressed in each cell subcluster were subsequently identified using the FindAllMarkers function, and cell subgroups were then classified according to the most abundantly expressed marker genes [16]. Tumor cells, T cells, macrophages, and fibroblasts were reintegrated and aggregated using the Seurat package. In this study, a second clustering was performed to identify clusters for each cell type. The second clustering followed the same process as the first clustering, with a resolution ranging from 1 to 7. Subsequently, the resulting cell types were annotated with cell subclusters based on representative/functional genes with significant specificity.

### 2.7 Functional Enrichment

Kyoto Encyclopedia of Genes and Genomes (KEGG) pathway enrichment analyses were performed using the clusterProfiler software package [17] to determine the potential function of the molecular pathways occurring in each cellular subpopulation. Pathways were considered significantly associated with marker genes when their *P* values were adjusted to <0.05.

### 2.8 Pseudotime Analysis

We utilized the Monocle3 software package to infer the developmental trajectories of cells. Initially, the integrated data's Seurat object was transformed into a cell_data_set object for compatibility with Monocle3 [18]. Subsequently, Monocle3 performed cell reclustering, assigning them to specific clusters and partitions, and then used these clusters and partitions to construct trajectories. Pseudotime, representing the distance between cells along the trajectory and the starting cell, was computed during the trajectory learning process. These computations were accomplished through the clustercells and learngraph functions in Monocle3. The plot_cells function in Monocle3 was employed to generate trajectory plots, overlaying trajectory information onto the UMAP representation of the integrated data. By adjusting the label_principal_points parameter, the names of root, leaf, and branch points could be displayed. During trajectory analysis in Monocle3, several nodes were identified and marked on the resultant plot with black circular markers, representing principal nodes along the trajectory. To determine the order of cells and compute their corresponding pseudotime values, it was necessary to select a starting node within the identified principal nodes and then sort cells and assign pseudotime values using the order_cells function in Monocle3. The pseudotime information obtained could be visually displayed on the UMAP plot using the plot_cells function, and users could extract pseudotime values of cells from the cell_data_set object using Monocle3's pseudotime functions. These pieces of information could be stored in the metadata of the Seurat object for further analysis.

### 2.9 Gene Regulatory Networks Analysis

To identify the internal transcriptional regulatory drivers of THCA we analyzed and reconstructed the gene regulatory network centered on transcription factors (TFs) [16] using the Python module tool pySCENIC [19].

The workflow begins by describing an input single-cell expression level profile matrix and then uses a regression approach for each target to infer co-expression modules, thereby identifying tailored indirect targets based on findings of cis-regulatory patterns. Subsequently, AUcell was used to quantify the activity of these regulators by enriching and scoring their target genes to obtain a regulon activity score (RAS). Single-cell 0data were further narrowed using the RAS matrix, and regulon-specific scores (RSS) were calculated according to the Jensen-Shannon divergence to identify cell cluster-specific regulators. The most specific and important regulators were mapped onto the single-cell cluster atlases and validated using massively parallel sample sequencing. Finally, the Connection Specificity Index (CSI) matrix was calculated, the regulators were hierarchically clustered according to the CSI, and the regulator modules were defined to identify the relationship between regulator modules and regulators. These relationships were then visualized using the R package ComplexHeatMap [20].

### 2.10 Intercellular Communication Analysis

We employed iTALK [21, 22] to elucidate receptor-ligand interactions among our delineated cell subpopulations and immune cells, thereby delineating cell-cell communication pathways. iTALK categorizes ligand-receptor pairs into four distinct groups: cytokines, growth factors, immune checkpoints, and others. In our current investigation, we implemented a two-step filtering approach based on cell inclusion criteria: firstly, we excluded follicular epithelial cells originating from normal thyroid tissue, while inclusively considering all malignant epithelial cells derived from tumor tissues. Secondly, we included immune cells and stromal cells as identified in the original study, namely: “NKT”, “CD8^+^ T”, “CD4^+^ T”, “macrophages”, “Mono”, “Naive.T”, “EN”, “LyEN”, and “Fibroblasts”. We opted to present the initial 25 receptor-ligand pairs, given that chordal plots offer a more visually impactful representation.

### 2.11 Molecular Docking

Possible protein-mRNA binding sequences were predicted using catRAPID omics v2.0 [23]. Molecular docking was performed using Hex 8.0.0 [24] to test the feasibility of ligand-receptor binding in THCA. Docking models were visualized using Pymol [25]. Docking energies less than 0 indicate that the two sequences have the potential to bind, and smaller energies indicate greater binding potential.

## 3 RESULTS

### 3.1 Single-cell Expression Profiles of Thyroid Cancer Ecosystems

**Fig. (1):**
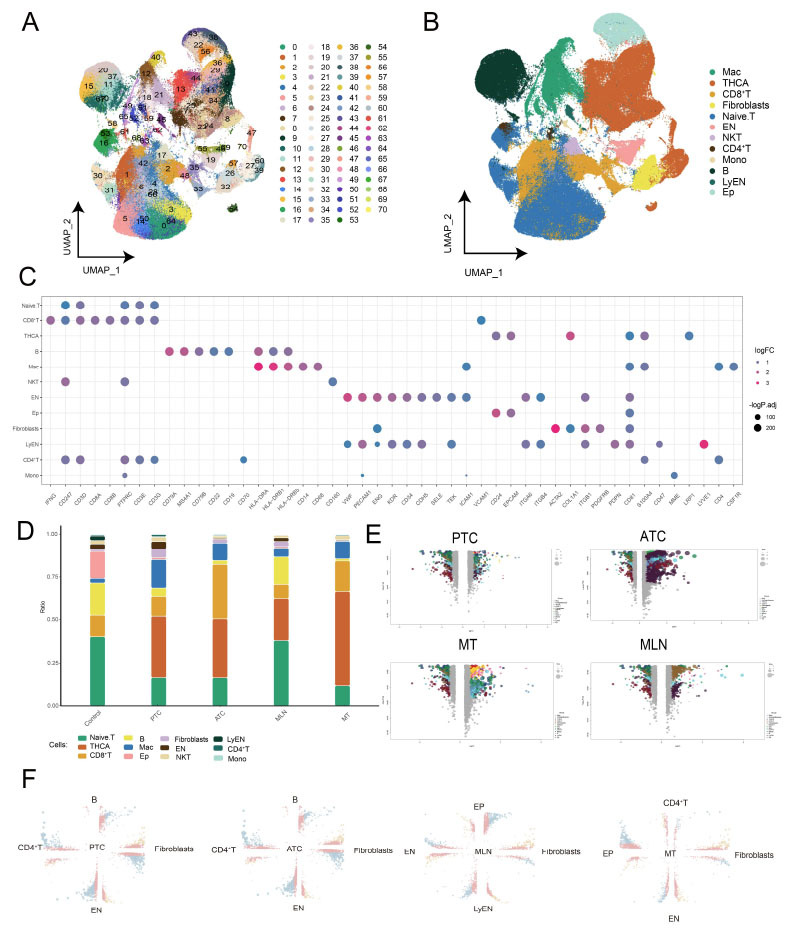
Single-cell expression profiles of thyroid cancer ecosystems. (**A**) A total of 24,9738 cells and 53 clusters were obtained to construct the panoramic single-cell atlas of THCA. (**B**) The 53 clusters were grouped into different cell types based on known marker genes, with each color representing a different cell type. These include macrophages (Mac), thyroid cancer cells (THCA), CD8^+^ T, fibroblasts (Fibroblast), naive T cells (Naive.T), endothelial cells (EN), natural killer T cells (NKT), CD4^+^ T, monocytes (Mono), B cells (B), lymphatic vessel endothelial cells (LyEN), and epithelial cells (Ep). (**C**) Bubble diagram showing specific marker genes in the different cell types. (**D**) Stacked bar graph showing changes in various cell fractions in the control, THCA patient tissue, lymph node metastasis, and distant metastasis groups. (**E**) Identifying differentially expressed genes for each cell type in various groups, compared to the control group. In PTC, ATC, MLN, and MT groups, genes with differential expression are represented by different colors. The left side indicates genes with low expression, while the right side indicates genes with high expression. (**F**) Identifying differentially expressed genes for each cell type in different groups compared to the control group. In the PTC, ATC, MLN, and MT groups, genes differentially expressed in each cell type are shown, with genes with low expression on the left and genes with high expression on the right. UMAP: uniform manifold approximation and projection.

Herein, we aimed to comprehensively investigate the heterogeneity of the tumor ecosystem during THCA initiation and progression. We analyzed a total of 23,207 single cells from 6 paracancerous tissues, 11 primary tumors, 8 MLN tissues, and 2 MT, using scRNA-seq technology (10×Genomics) (Fig. **1A**). We map the sample sources. (Supplementary Fig. **1A**).

By integrating the transcriptional data of all obtained cells, we constructed a single-cell expression profile of THCA. Using UMAP clustering, we identified six major cell populations: T cells, B cells, thyroid cancer cells, macrophages, fibroblasts, and endothelial cells. To gain a more detailed understanding of the immune landscape, we performed UMAP analysis and further classified T cells into subpopulations based on their differentially expressed genes. We specifically identified CD4^+^ T cells, CD8^+^ T cells, and Naive T cells, based on their expression levels of CD4 and CD8 (Fig. **1B**). The cell populations were defined based on the expression of specific marker genes associated with each cell type (Fig. **1C**). We similarly mapped the sample sources for each cell type (Supplementary Figs. **1B-F**). Later we observed the proportions of these cell types in different subgroups (Fig. **1D**). We additionally identified differentially expressed genes for each cell type across different groups, relative to the control group (Figs. **1E**, **F**).

Furthermore, we found tumor-specific clustering among malignant cells, indicating a high degree of inter-tumor heterogeneity. By identifying signature genes for each tumor cell subcluster, we successfully developed four tumor cell subtype-specific signatures. These findings provide valuable insights into the cellular heterogeneity and differentially expressed genes within thyroid tissue from patients with THCA, which might contribute to further characterizing clinical staging and understanding the tumor microenvironment.

In summary, through the construction of a global single-cell atlas of thyroid tissue from patients with THCA, we determined the global cellular ecological differences and identified inter- and intra-tumor cell-specific genes. These findings will contribute to our understanding of THCA heterogeneity and provide potential markers for clinical characterization and staging of thyroid cancer.

### 3.2 IGF2BP3 and VIRMA may Enhance Malignant Proliferation and Invasion in ATC by Upregulating lncRNA XIST

**Fig. (2):**
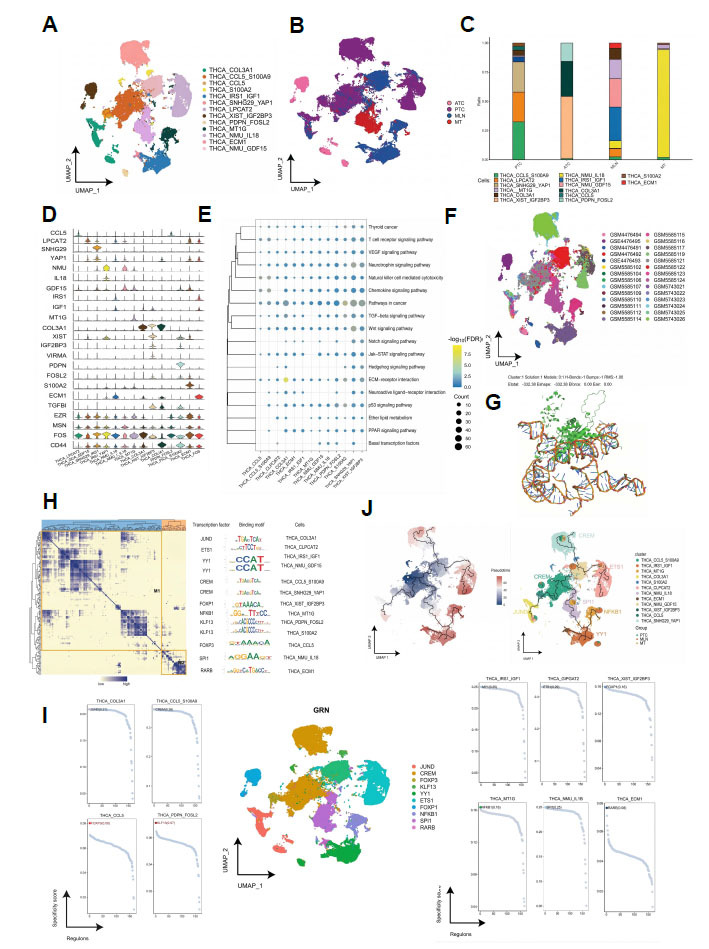
IGF2BP3 and VIRMA may enhance malignant proliferation and invasion in ATC by upregulating lncRNA XIST. (**A**) Single-cell atlas of THCA cell subpopulations; each color represents a different subpopulation. (**B**) Single-cell mapping of different subpopulation cell mapping groupings. (**C**) Differences in the abundance of THCA cell subpopulations in the different subgroups. (**D**) The expression of marker genes in each subpopulation. (**E**) Bubble diagram showing biological pathways of the THCA cell subpopulations. (**F**) Single-cell atlas of mapped sample sources. (**G**) The molecular docking of lncRNA XIST to IGF2BP3 with an shape of -332.38. (**H**) Co-expression modules of transcription factors in THCA cells. Left: The identification of regulatory modules based on their CSI matrices; middle: The binding patterns of representative transcription factors in the modules; right: The cellular subpopulations in which the transcription factors are located. (**I**) UMAP single-cell atlas plots of specific GRNs for subpopulations of THCA cells. CSI: linkage specificity index; UMAP: uniform manifold approximation and projection; GRN: gene regulatory network. (**J**) Single-cell atlas plotting the trajectory and pseudo-time values of THCA progression. Pie charts show the proportion of different groups in the clusters.

We aimed to identify and characterize major subpopulations of thyroid cancer cells based on the expression of characteristic genes. We identified a total of 13 distinct subpopulations of thyroid cancer cells (Supplementary Fig. **1G**). We conducted a detailed analysis wherein THCA samples were meticulously annotated into PTC and ATC subtypes, leveraging the distinct expression patterns of marker genes (Fig. **2A**). Furthermore, through comprehensive single-cell mapping, we juxtaposed thyroid differentiation genes, revealing a notable enrichment within the PTC subgroup. This observation strongly suggests that ATC exhibits a heightened degree of dedifferentiation compared to PTC, underscoring the divergent molecular landscapes inherent to these thyroid cancer subtypes (Supplementary Fig. **2**). Further investigation into the abundance of these cell clusters in patients with THCA revealed interesting patterns. The THCA_SNHG29_YAP1 subpopulation was found to be more abundant in patients with PTC, while the THCA_XIST_IGF2BP3 subpopulation was more abundant in patients with ATC. Additionally, the THCA_NMU_IL18 subpopulation was more abundant in patients with MT (Figs. **2B**, **C**). We further investigated the marker genes associated with different clusters of THCA cells and visualized their expression using a violin plot. Some of the markers included SNHG29 (a long noncoding RNA (lncRNA), small nucleolar RNA host gene 29), YAP1 (encoding yes associated protein 1), XIST (X inactive specific transcript, a lncRNA), IGF2BP3 (encoding insulin-like growth factor 2 mRNA binding protein 3), and NMU (encoding neuromedin U), among others (Fig. **2D**). Interestingly, we observed that the THCA_SNHG29_YAP1 and THCA_XIST_IGF2BP3 cell clusters were predominantly derived from individual patients. Additionally, we examined the co-expression of the lncRNA SNHG29 and YAP1, as well as lncRNA XIST and IGF2BP3 (Supplementary Figs. **3A, B**). Enrichment analysis revealed that these two subpopulations were closely associated with cancer pathways. (Fig. **2E**). To explore the diversity of THCA, we mapped the sample sources of tumor cells and found significant patient heterogeneity among the tumor samples (Fig. **2F**). Intriguingly, we found that IGF2BP3,lncRNA XIST, and VIRMA (encoding Vir-like m6A methyltransferase associated) were expressed in the same ATC subpopulation (Supplementary Figs. **3C, D**). We hypothesized that IGF2BP3 and VIRMA might promote the malignant proliferation and invasion of ATC by increasing the expression of the lncRNA XIST. To support this hypothesis, we performed molecular docking analysis of lncRNA XIST and IGF2BP3, which showed the energy value was -332.38 kJ/mol. Docking energy < 0 kj/mol indicated a binding potential, suggesting a potential binding interaction between IGF2BP3 and lncRNA XIST. (Fig. **2G**). We also tested METTL14 with lncRNA XIST, which was confirmed in the previous article [26], and the result energy value was -708.85kj/mol (Supplementary Fig. **1J**). While the energy value of IGF2BP3 binding to XIST may be smaller than that of METTL14 binding to XIST, this does not necessarily rule out the possibility of the former binding. Furthermore, we constructed GRNs of the THCA subpopulations using TFs such as forkhead box P1 (FOXP1) and others. These TFs organized the GRNs into two modules, regulating the specific gene expression in THCA cells (Figs. **2H**, **2I**). Notably, the transcription factor FOXP1 was associated with the THCA_XIST_IGF2BP3 subpopulation. Previous studies have reported that upregulated lncRNA XIST promotes tumor cell proliferation, autophagy inhibition, and apoptosis suppression by inducing FOXP1 overexpression [27].

Finally, by performing temporal analysis of PTC cells, we inferred that the THCA_CCL5_S100A9 cluster served as the starting point of development, evolving into THCA_NMU_IL18, THCA_LPCAT2, and THCA_IRS1_IGF1 subpopulations (Fig. **2J**). In conclusion, our findings indicate that IGF2BP3 and VIRMA may enhance the upregulation of the lncRNA XIST gene, thereby facilitating the malignant progression of cancer cells. These insights provide valuable information about the potential molecular mechanisms underlying thyroid cancer development and progression.

### 3.3 ATC Might Promote Tumor Aggressiveness by Reprogramming CD4^+^T Cells

**Fig. (3):**
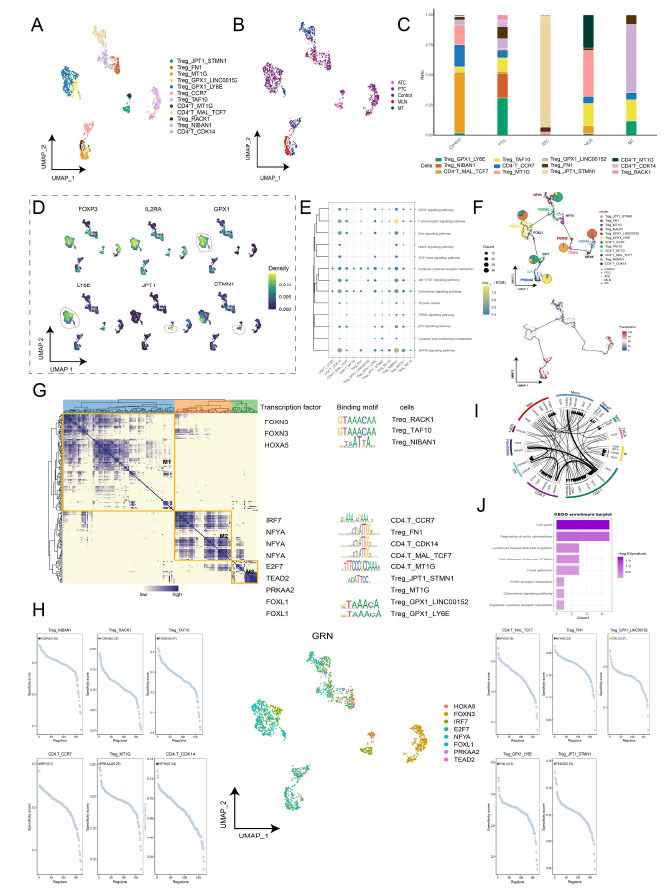
ATC might promote tumor aggressiveness by reprogramming CD4^+^T cells. (**A**) Single-cell atlas of CD4^+^ T cell subpopulations; each color represents a different subpopulation. (**B**) Single-cell atlas showing the different groupings of cell subpopulations. (**C**) Differences in the abundance of CD4^+^ T cell subpopulations in the different groupings. (**D**). The expression of marker genes in the main subpopulation. (**E**) Bubble diagram showing the biological pathways of CD4^+^ T cell subpopulations. (**F**) single-cell atlas plotting the trajectory of CD4^+^ T progression and pseudo-time values. Pie charts show the proportion of the different populations in the cluster. (**G**) Co-expression modules of the transcription factors in CD4^+^ T cells. Left: The identification of regulatory modules based on their CSI matrices; middle: The binding patterns of representative transcription factors in the modules; right: The cellular subpopulations in which the transcription factors are located. (**H**) UMAP single-cell mapping of CD4^+^ T cell subpopulations with specific GRNs. (**I**) The figure shows an immune checkpoint module of the cellular communication network demonstrating communication between individual cell types in ATC groupings, such as intercellular communication between tumor cells and CD4^+^T cells *via* the CD70-CD27 axis. (**J**) The figure demonstrates the enrichment of Treg_JPT1_STMN1 in the KEGG pathway, which showed that this subpopulation was associated with the cell cycle, actin skeleton regulation, and leukocyte migration.

Tumor cells can reprogram their surrounding immune microenvironment, thereby promoting their proliferation and migration. Herein, we re-clustered the CD4^+^ T cells in THCA and identified 12 distinct cell subgroups (Fig. **3A**). CD4^+^ T cells co-expressing FOXP3 and IL2RA (encoding interleukin 2 receptor subunit alpha) were annotated as Treg cells. Single-cell profiles provided insights into the CD4^+^ T cell subpopulations within different subgroups (Fig. **3B**). We observed that Tregs were enriched in tumor tissue compared with paraneoplastic samples (Fig. **3B**).

Comparing patients with PTC to those with ATC, ATC triggers an aggressive and immunosuppressive microenvironment characterized by specific enrichment of the Treg_JPTI_STMN1 subpopulation in ATC. (Fig. **3C**). Single-cell density profiles also demonstrated the expression of the STMN1 gene (encoding stathmin 1) and the JPT1 gene. (Fig. **3D**). Jupiter microtubule-associated homolog 1 (JPT1), also known as hematological and neurological expression 1 (HN1), first identified in mouse embryos, is an evolutionarily conserved protein, and existing studies have shown that JPT1 is involved in cell growth, repair, and regeneration [28]. STMN1 is overexpressed in a variety of tumors and is closely associated with tumor growth, metastasis, and immune infiltration [29, 30]. It has been shown that JPT1 promotes STMN1 expression and reduces STMN1 degradation. We identified a subpopulation of Treg cells that specifically highly expressed JPT1 and STMN1 in ATC, and we enriched this subpopulation for the KEGG pathway, which showed that this subpopulation was associated with the cell cycle, actin skeleton regulation, and leukocyte migration (Fig. **3J**). We hypothesized that JPT1 and STMN1 interaction promotes Treg cell proliferation and migration to the tumor, and promotes the formation of an ATC-immunosuppressive tumor microenvironment.

Enrichment analysis revealed that Treg cell subclusters significantly activated protein kinase (MAPK)-related signaling pathways (Fig. **3E**). These signaling pathways play important roles in immune responses. The proposed temporal sequence analysis demonstrated the developmental trajectory of CD4^+^ T cells, starting from CD4^+^T_MAL_TCF7 and progressing to other subpopulations (Fig. **3F**). Furthermore, we constructed a GRN and identified three modules regulated by TFs such as FOXP1 and TEA domain transcription factor 2 (TEAD2). These TFs are involved in regulating the specific gene expression in CD4^+^ T cells (Figs. **3G**-**3H**).

We observed in the immune checkpoint module of the cellular communication network that tumor cells expressed only CD70, while immune cells, such as CD4^+^ T cells, expressed both CD70 and CD27. Cellular communication between CD27 on immune cells and CD70 on tumor cells suggests the presence of a CD70-CD27 axis in ATC cells (Fig. **3I**).

### 3.4 Enrichment of CD8^+^ T Cells Expressing PDCD1 in ATC

**Fig. (4):**
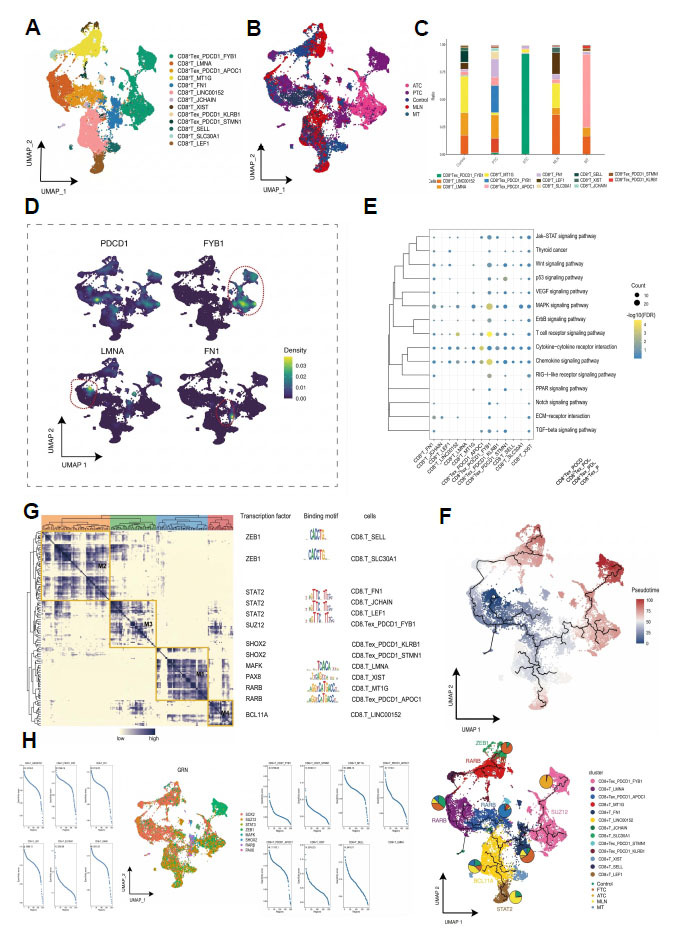
Enrichment of CD8^+^ T cells expressing PDCD1 in ATC. (**A**) Single-cell atlas of CD8^+^ T cell subpopulations; each color represents a different subpopulation. (**B**) Single-cell atlas showing the different groupings of cell subpopulations. (**C**) Differences in the abundance of CD8^+^ T cell subpopulations in the different groupings. (**D**) The expression of marker genes in the main subpopulation. (**E**) Bubble diagram showing the biological pathways of CD8^+^ T cell subpopulations. (**F**) Single-cell atlas plotting the trajectory of CD8^+^ T progression and pseudo-time values. Pie charts show the proportion of the different populations in the clusters. (**G**) Co-expression modules of the transcription factors in CD8^+^ T cells. Left: The identification of regulatory modules based on their CSI matrices; middle: The binding patterns of representative transcription factors in the modules; right: The cellular subpopulations in which the transcription factors are located. (**H**). UMAP single-cell mapping of CD8^+^ T cell subpopulations with specific GRNs.

To identify distinct cell subpopulations, CD8^+^ T cells were re-clustered, resulting in the identification of 12 different subpopulations (Fig. **4A**). Single-cell profiles revealed the presence of CD8^+^ T cell subpopulations within different subgroups (Fig. **4B**). We annotated CD8^+^ T cells co-expressing PDCD1 (encoding programmed cell death 1) as depleted T cells (exhausted T cells, Tex) [31]. We also observed that Tex cells were enriched in tumor tissue compared to paraneoplastic samples, suggesting that tumor cells in the THCA microenvironment influence the immune system and facilitate immune escape (Fig. **4C**). Notably, the abundance of the CD8^+^T_PDCD1_FYB1 subpopulation was significantly increased in patients with ATC (Fig. **4C**). Additionally, single-cell density profiles revealed the expression of certain marker genes (Fig. **4D**).

Enrichment analysis revealed that CD8^+^ T cell subclusters were significantly associated with activating MAPK-related signaling pathways, particularly in the CD8^+^T_PDCD1_FYB1 subpopulation (Fig. **4E**). The MAPK pathway has been shown to promote cell survival, proliferation, and immune escape in tumors by inhibiting cell death signaling and upregulating PDCD1 expression [32]. However, Further validation is needed to determine the association between CD8^+^ T cells' enrichment of PDCD1 in ATC and the MAPK pathway.

The proposed chronological analysis demonstrated the developmental trajectory of CD8^+^ T cells, starting from CD8^+^T_LMNA and progressing to other subpopulations (Fig. **4F**). Furthermore, the GRN analysis identified three CD8^+^ T cell population modules, regulated by TFs such as zinc finger BED-type containing 1 (ZBED1) and paired box 8 (PAX8). These modules controlled the specific gene expression in CD8^+^ T cells (Figs. **4G**, **4H**).

In conclusion, the upregulation of PDCD1 expression in CD8^+^ T cells might be influenced by the MAPK pathway, leading to the immune escape of tumor cells and the depletion of CD8^+^ T cells.

### 3.5 The Joint Action of GPNMB and CCL18 on Macs Might Promote Tumor Cell Invasion.

**Fig. (5):**
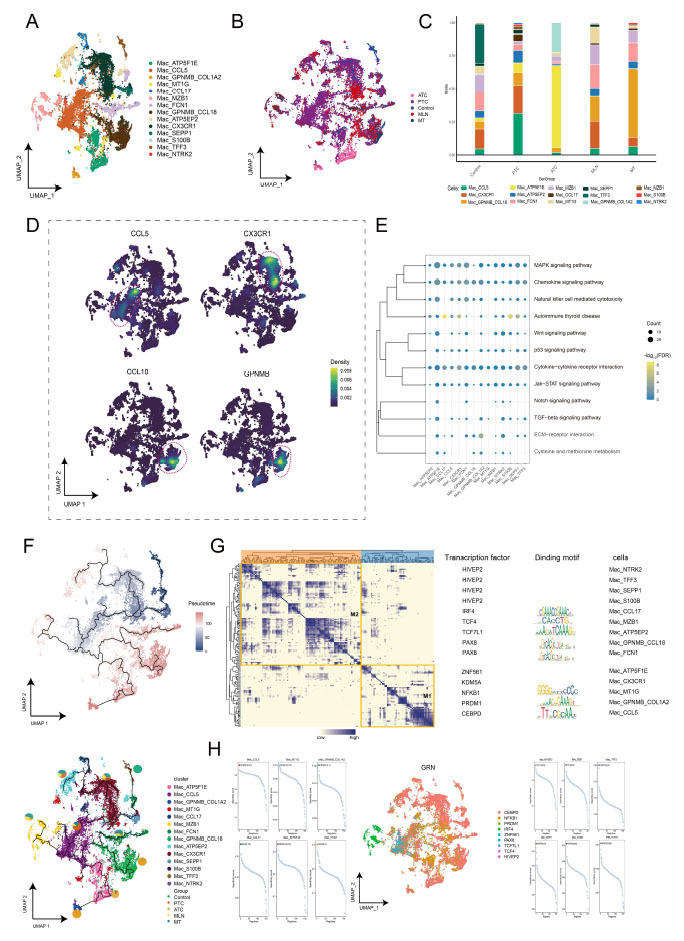
The joint action of GPNMB and CCL18 on Macs might promote tumor cell invasion. (**A**) Single-cell atlas of Mac subpopulations; each color represents a different subpopulation. (**B**) Single-cell atlas showing the different groupings of cell subpopulations. (**C**) Differences in the abundance of Mac subpopulations in the different groupings. (**D**) Expression of marker genes in the main subpopulation. (**E**) Bubble diagram showing the biological pathways of Mac subpopulations. (**F**) Single-cell atlas plotting trajectories of Mac progression and pseudo-time values. Pie charts show the proportion of different populations in the cluster. (**G**) Co-expression modules of transcription factors in Macs. Left: The identification of regulatory modules based on their CSI matrices; middle: The binding patterns of representative transcription factors in the modules; right: The cellular subpopulations in which the transcription factors are located. (**H**) UMAP single-cell mapping of specific GRNs in Mac subpopulations.

To identify distinct cell subpopulations, we performed a re-clustering of Macs, resulting in the identification of 15 different subpopulations (Fig. **5A**). Single-cell profiles revealed the presence of various subgroups within the Mac population (Fig. **5B**). In Fig. (**5C**), the bar graph illustrates the proportions of different macrophage subpopulations in each subgroup. Additionally, single-cell density profiles depict the expression of marker genes CCL18 (encoding C-C motif chemokine ligand 18), and GPNMB (encoding glycoprotein Nmb) (Fig. **5D**). It is worth noting that GPNMB has immunosuppressive effects in malignant tumors [33]. Macrophages with high expression of GPNMB promote tumor progression and inhibit T-cell activation [34]. GPNMB can also induce M2 polarization in macrophages [35]. Furthermore, CCL18 is upregulated in tumor-promoting M2 macrophages, facilitating tumor cell invasion and metastasis [35].

Enrichment analysis revealed that Mac subclusters were significantly associated with extracellular matrix (ECM) receptor interactions and MAPK-related signaling pathways. Moreover, these subclusters showed a close association with autoimmune thyroid disease (Fig. **5E**). The GRN analysis of Mac clusters indicated the division of marker genes into two modules, regulated by TFs such as PAX8, and CCAAT enhancer binding protein delta (CEBPD). Furthermore, we constructed the GRN of Macs, in which the GRN was identified by TFs such as PAX8, and CEBPD and organized into two modules (Figs. **5F**, **G**). These modules exerted regulatory control over the specific gene expression in Macrophages.

The proposed temporal analysis suggested that the developmental trajectory of Macs evolves from Mac_TFF3 as the starting point, progressing to other subpopulations (Fig. **5H**).

### 3.6 Enrichment of Tumor-associated Fibroblasts in Thyroid Cancer

**Fig. (6):**
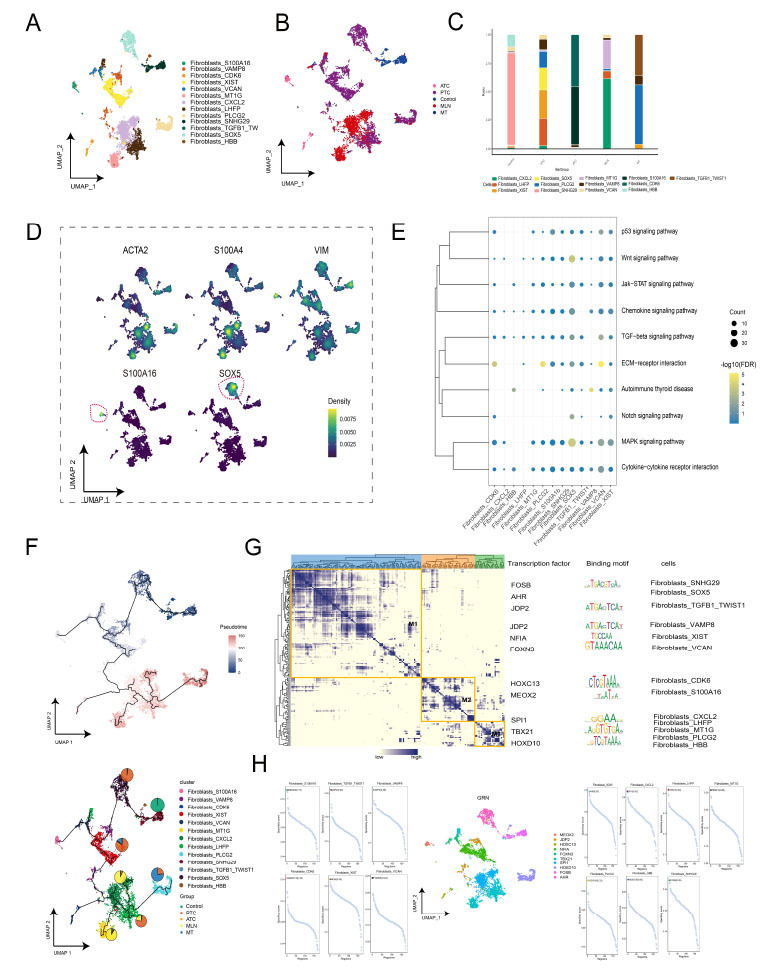
Enrichment of tumor-associated fibroblasts in thyroid cancer. (**A**) Single-cell atlas of Fibroblast cell subpopulations; each color represents a different subpopulation. (**B**) Single-cell atlas showing different subgroups of cell subpopulations. (**C**) Differences in the abundance of fibroblast cell subpopulations in the different subgroups. (**D**) Expression of marker genes in the main subpopulation. (**E**) Bubble plots showing the biological pathways of fibroblast cell subpopulations. (**F**) Single-cell atlas plotting the trajectory and pseudo-time values of fibroblast progression. Pie charts show the proportion of the different populations in the cluster. (**G**) Co-expression modules of transcription factors in fibroblast cells. Left: The identification of regulatory modules based on their CSI matrices; middle: The binding patterns of representative transcription factors in the modules; right: The cell subpopulations in which the transcription factors are located. (**H**) UMAP single-cell mapping of fibroblast cell subpopulations with specific GRNs.

Tumor cells can activate fibroblasts *via* secretory factors, leading to their transformation into tumor-associated fibroblasts (CAFs), which interact with tumor cells to promote the progression of thyroid cancer [36]. We performed a re-clustering of fibroblast cells, resulting in the identification of 13 distinct cell subpopulations (Fig. **6A**). Single-cell profiles demonstrated the presence of fibroblast cell subpopulations within different subgroups (Fig. **6B**). (Fig. **6C**) shows bar graphs illustrating the proportions of different fibroblast subpopulations in each subgroup, while single-cell density profiles depict the expression of marker genes (Fig. **6D**). We annotated fibroblasts co-expressing ACTA2 (encoding actin alpha 2, smooth muscle), S100A4 (encoding S100 calcium-binding protein A4), and VIM (encoding vimentin) as tumor-associated fibroblasts because nearly all fibroblasts exhibited co-expression of these three markers.

Moreover, we found that fibroblast cell subclusters were significantly enriched in transforming growth factor beta (TGFb) signaling, and MAPK-related signaling pathways, as revealed by enrichment analysis. These subclusters were also closely associated with autoimmune thyroid disease (Fig. **6E**). Chronological analysis demonstrated the developmental trajectory of fibroblast cells, originating from Fibroblast_SNHG29 and progressing to other subpopulations. Overall, the proposed temporal analysis supports the notion that fibroblast cells undergo developmental changes, with Fibroblast_SNHG29 serving as the starting point and giving rise to other subpopulations (Fig. **6F**). Additionally, we constructed the GRN of fibroblast cells, including TFs such as FOXN3 and others. The GRN was organized into three modules Figs. (**6G**, **H**), with each module regulating specific gene expression in fibroblast cells. 

## 4 DISCUSSION

In this study, we aimed to establish a comprehensive single-cell landscape of thyroid cancer lesions. We sought to uncover the heterogeneity of thyroid cancer at the single-cell level and explore the variations in gene expression within tumor cells and the tumor microenvironment across different pathological stages. By doing so, we aimed to gain insights into the complex multicellular ecosystem of thyroid cancer.

We mapped the sample sources of thyroid cancer cells and observed that cells from different pathological types or stages exhibited specific gene expression profiles. As mentioned above, we found that the THCA_SNHG29_YAP1 subpopulation was enriched in PTC (Fig. **2C**), YAP1 (Yes-associated protein 1) is a crucial transcriptional co-activator that translocates to the nucleus when maintained in a dephosphorylated state. In this state, it binds to TEAD (TEA-domain family) transcription factors, stimulating cell growth, proliferation, and survival. Prior research has demonstrated that in colorectal cancer cells, lncRNA SNHG29 is capable of sustaining the dephosphorylated state of YAP1, thereby enhancing cancer cell proliferation. This implies that both lncRNA SNHG29 and YAP1 may play significant roles in the onset and progression of PTC. Specifically, we found higher expression levels of the lncRNA XIST and IGF2BP3 in ATC. IGF2BP3 functions as an m6A “reader” and has been reported to stabilize mRNA by preventing mRNA degradation through recognition of m6A modifications [37]. The lncRNA XIST has been implicated in promoting thyroid cancer growth and invasion [38]. Notably, lncRNA XIST is enriched with m6A sites, and m6A modification plays a crucial role in regulating the stability and function of XIST. Specifically, this modification enhances the stability of XIST and is associated with its role in gene silencing on the -X chromosome [39]. F Additionally, VIRMA acts as an m6A “encoder” [40] and may contribute to the stability of lncRNA XIST through m6A modification. Based on these findings, we hypothesized that IGF2BP3 and VIRMA might inhibit the degradation of lncRNA XIST by recognizing its m6A modification, thereby promoting the growth and invasion of thyroid cancer. Based on these findings, we hypothesized that IGF2BP3 and VIRMA might inhibit the degradation of lncRNA XIST by recognizing its m6A modification, thereby promoting the growth and invasion of thyroid cancer. Moreover, previous studies have indicated that lncRNA XIST is exclusive to the X chromosome in females [41, 42], which might contribute to the higher incidence of thyroid cancer in females compared with males in clinical practice. We mapped the single-cell profile of lncRNA XIST to distinguish the sex of patients by the distribution of lncRNA XIST (Supplementary Figure **1I**).

In the tumor microenvironment of thyroid cancer, immune cells, and stromal cells undergo alterations that contribute to tumor development. Our analysis revealed enrichment of Treg and Tex cells in the tumor groups compared with those in the controls. CD4^+^ T cells overexpress JPT1 and STMN1, which promote ATC invasion. JPT1 is upregulated in various cancers, including breast cancer and hepatocellular carcinoma, where it promotes malignant invasion. STMN1 is considered an oncogene that promotes the proliferation, invasion, and metastasis of cancer cells [43-45]. JPT1 has been shown to increase the expression of STMN1, and their interaction enhances the invasiveness of ATC cells [46]. In addition, we found a CD27-CD70 axis between ATC and the tumor microenvironment in cellular communication. CD27 is a transmembrane glycoprotein in the tumor necrosis factor receptor superfamily (TNFRSF), primarily expressed in T cells, B cells, NK cells, and some dendritic cells. It acts as a co-stimulatory molecule, crucial for immune response regulation. Its ligand, CD70, is expressed on activated immune cells and plays a transient role in immune responses. However, in pathological conditions, CD70 is overexpressed in tumors. Dysregulation of the CD70-CD27 axis further allows tumor cells to escape immune surveillance in the TME. This axis impedes the anti-tumor immune response by inducing T cell depletion, activating Tregs, and depleting NK cells [47]. In our study, we identified the CD70-CD27 axis in the intercellular communication of anaplastic ATC subgroups. Specifically, thyroid cancer cells established this axis with CD4^+^ T, CD8^+^ T, B, and fibroblasts, highlighting its role in immune evasion and immunosuppression in the TME of ATC.

## CONCLUSION

In conclusion, our study provides a comprehensive single-cell landscape of thyroid cancer, offering valuable insights into the complex ecosystem of THCA. However, certain limitations need to be acknowledged. Firstly, the inclusion of only 20 patients from the GEO database restricted the generalizability of our findings. Secondly, our study primarily focuses on molecular expression and relies on an experimental model. Therefore, further investigations utilizing cellular and animal experimental models are necessary to validate our findings and explore the molecular interactions in greater depth.

## AUTHORS’ CONTRIBUTIONS

The authors confirm their contributions as follows: JZ & XS: Conceptualization, Methodology KS & YW: Data Collection, Analysis, Writing Review, Visualization YW, JL & WY: Data Analysis, Writing JZ, YQ, YC & XS: Conceptualization, Supervision, Writing - Review & Editing.

All authors reviewed the results and approved the final version of the manuscript.

## Data Availability

The data used in this study are publicly available from the GEO (Gene Expression Omnibus) databases. (https://www.ncbi.nlm.nih.gov/geo/).

## LIST OF ABBREVIATIONS

PTC: Papillary Thyroid Carcinoma
MLN: Metastatic Lymph Nodes
PCa: Paracancerous Tissue
GRN: Gene Regulatory Networks
GEO: Gene Expression Omnibus
GLM: Generalized Linear Model
UMAP: Uniform Manifold Approximation and Projection
KEGG: Kyoto Encyclopedia of Genes and Genomes
