# Supplementary material for: Construction of a Single-cell Atlas of Thyroid Cancer
Source: Endocr Metab Immune Disord Drug Targets. 2025 Apr 3;26:E18715303359688. doi: 10.2174/0118715303359688250209090544 (PMC13284668; doi:10.2174/0118715303359688250209090544)
Supplement: Supplementary file 1 [file EMIDDT-26-E18715303359688_SD1.pdf]

Supplementary Material

Construction of a Single-cell Atlas of Thyroid Cancer

Kaiyu Song[1,#] , Yaqi Wang[2,#] , Yuantao Wang[3,#], Jiahui Liu[2,4,5,#] , Wenjie Yao[3] , Yongli Chu[6,*] , Yun Qu[7,*] , Xicheng Song[2,8,*] and Jin Zhou[1,8,*]

[1]Department of Endocrinology, Yantai Yuhuangding Hospital of Qingdao University, Yantai 264000, Shandong, China; [2]Department of Otorhinolaryngology, Head and Neck Surgery, Yantai Yuhuangding Hospital, Qingdao University, Yantai, China; [3]Department of Endocrinology, Binzhou Medical University, Yantai, Shandong, China; [4]Shandong Provincial Clinical Research Center for Otorhinolaryngologic Diseases, Yantai Yuhuangding Hospital, Qingdao University, Yantai, China; [5]Yantai Key Laboratory of Otorhinolaryngologic Diseases, Yantai, China; [6]Department of Gynecology, Yantai Yuhuangding Hospital of Qingdao University, Yantai, China; [7]Department of Emergency, Yantai Yuhuangding Hospital of Qingdao University, Yantai 264000, Shandong, China; [8]Key Laboratory of Spatiotemporal Single-Cell Technologies and Translational Medicine, Yantai 264000, Shandong, China

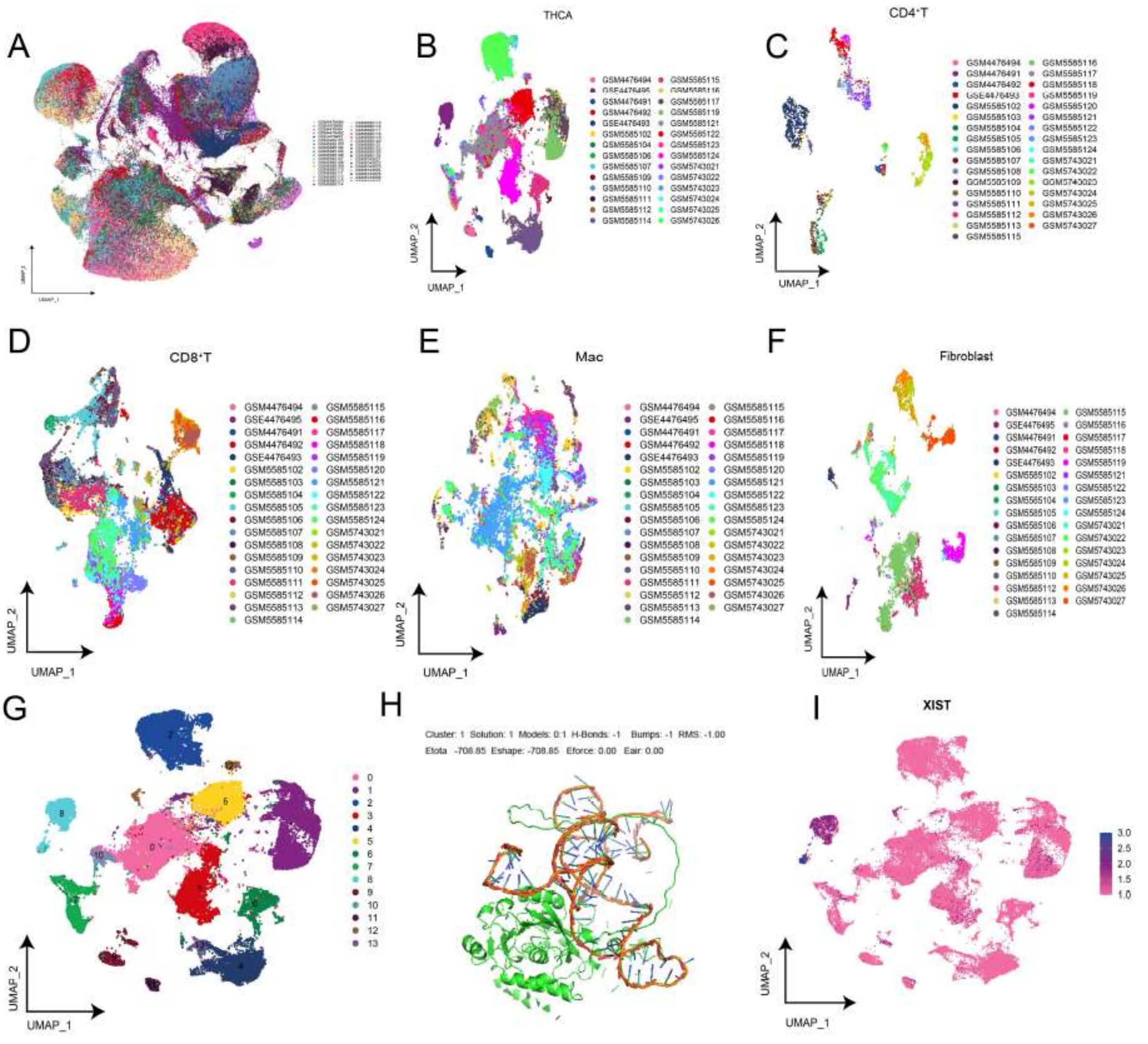

Supplementary Figure 1. (A) Mapping single-cell atlases from sample sources, with each color representing a different sample. (B) The single-cell atlas mapping of THCA cells based on sample origins; each color represents a different sample. (C) Mapping single-cell atlas of $CD4^+$ T cells based on sample origins; each color represents a different sample. (D) Mapping single-cell atlas of $CD8^+$ T cells based on sample origins; each color represents a different sample. (E) Mapping single-cell atlas of Mac cells based on sample origins; each color represents a different sample. (F) Mapping single-cell atlas of Fibroblast cells based on sample origins; each color represents a different sample. (G) Single-cell atlas of THCA subtypes, demonstrating the subdivision of thyroid cancer cells into 14 subgroups through dimensionality reduction clustering. (H) The molecular docking of lncRNA XIST to METTL14 with an eshape of -708.85. (I) The single-cell atlas shows the distribution of XIST in thyroid cancer cells.

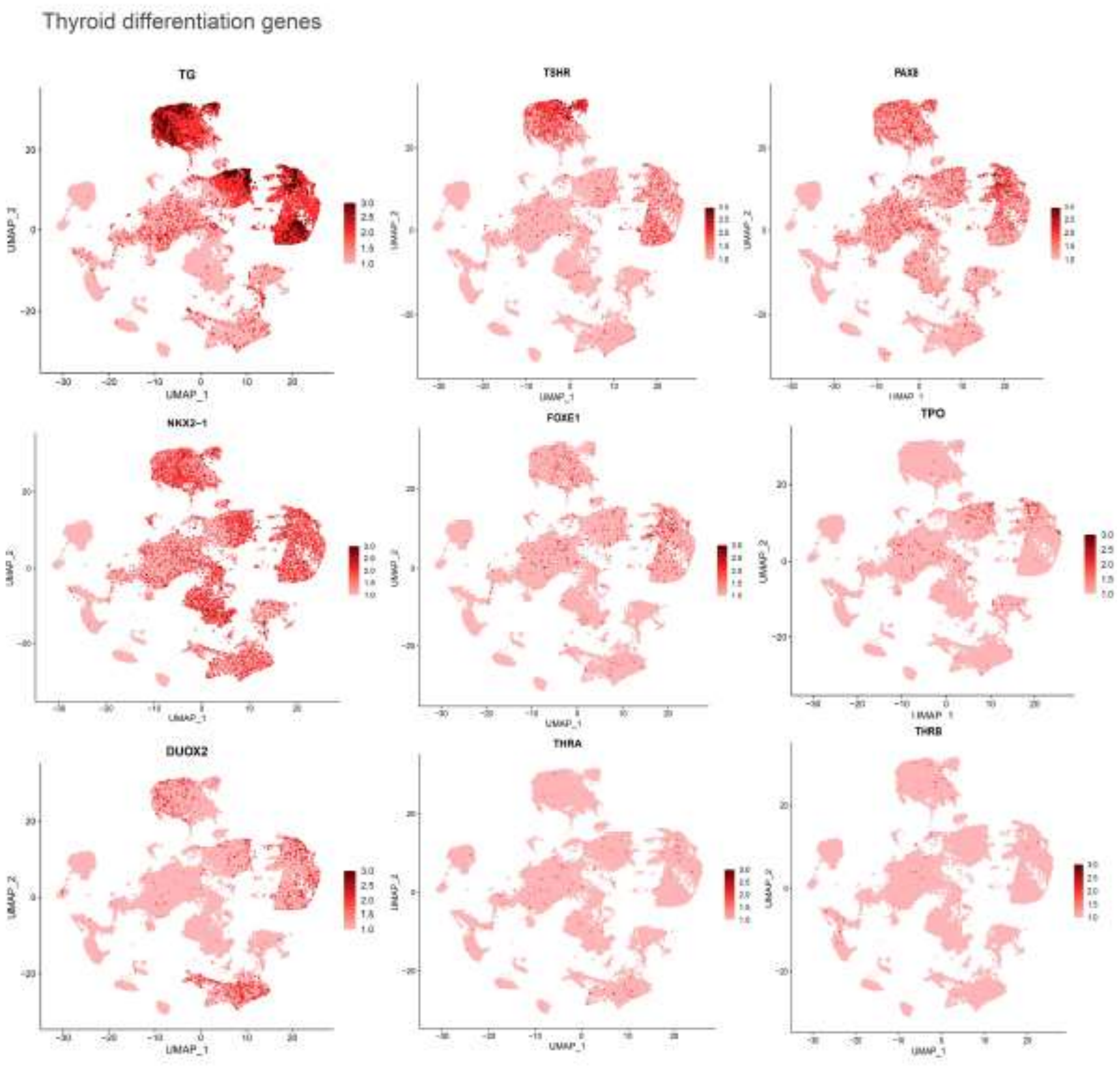

Supplementary Figure 2. The supplementary figure 2 presents single-cell atlases of 9 genes related to thyroid cancer differentiation.We can found that thyroid differentiation genes were more enriched in PTC, implying that ATC showed a higher degree of dedifferentiation.

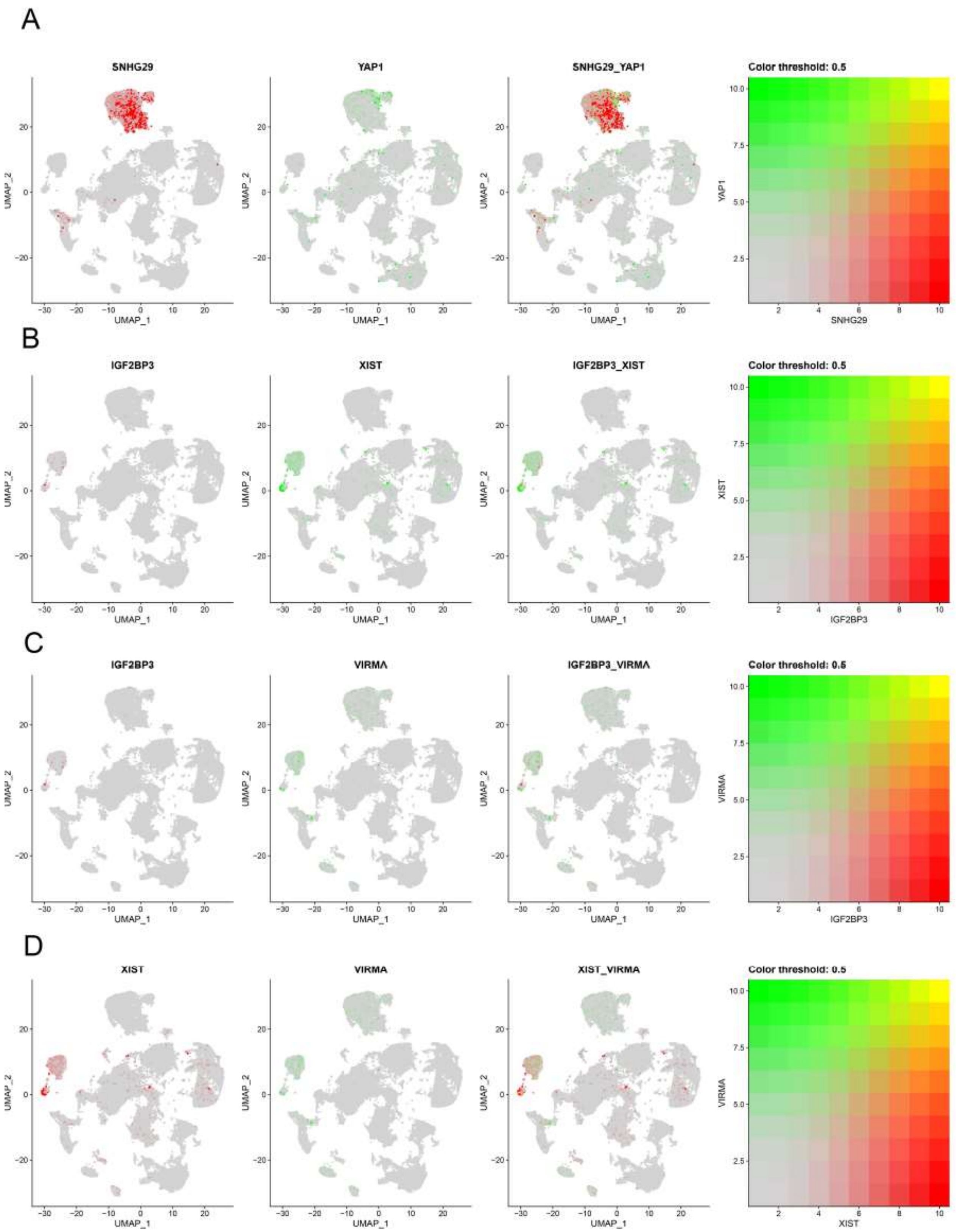

Supplementary Figure 3. (A) Figure A demonstrates the co-expression of SNHG29 and YAP1. (B) Figure B demonstrates the co-expression of IGF2BP3 and XIST. (C) Figure C demonstrates the co-expression of IGF2BP3 and VIRMA. (D) Figure D demonstrates the co-expression of XIST and VIRMA.
